# Four new species of *Phrynidius* Lacordaire (Coleoptera, Cerambycidae, Lamiinae) from Mexico with an identification key for the genus

**DOI:** 10.3897/zookeys.1000.56757

**Published:** 2020-12-03

**Authors:** Nayeli Gutiérrez, Víctor H. Toledo-Hernández, Felipe A. Noguera

**Affiliations:** 1 Richard Gilder Graduate School, American Museum of Natural History. Central Park West & 79th St, New York, NY 10024, USA American Museum of Natural History New York United States of America; 2 Centro de Investigación en Biodiversidad y Conservación, Universidad Autónoma del Estado de Morelos, Av. Universidad 1001, Col. Chamilpa, Cuernavaca, Morelos 62209, México Universidad Autónoma del Estado de Morelos Cuernavaca Mexico; 3 Estación de Biología Chamela, Instituto de Biología, Universidad Nacional Autónoma de México, Apartado Postal 21, San Patricio, Jalisco 48980, México Universidad Nacional Autónoma de México Jalisco Mexico

**Keywords:** Biodiversity, Central America, longhorn beetles, taxonomy

## Abstract

Since the description of its eight species, the Mesoamerican genus *Phrynidius* Lacordaire (Cerambycidae, Lamiinae, Apomecynini) has not been comprehensively studied, with only a few distributional records published in recent years. In this work, four new species of *Phrynidius* are described from Chiapas, Mexico: *P. cristinae***sp. nov.** from the municipality of Escuintla, *P. diminutus***sp. nov.** from San Cristobal, *Phrynidius jonesi***sp. nov.** from Trinitaria, and *P. tuberculatus***sp. nov.** from Jaltenango. An updated taxonomic key and illustrations of the new species are also provided.

## Introduction

Currently, *Phrynidius* Lacordaire, 1869 comprises eight apterous species (Tavakilian and Chevillotte 2020). The species size varies from 6 to 12 mm, with a camouflage pattern that resemble small rocks due to their dark, somber color and elevated tubercles spread on dorsal surface.

Studies on *Phrynidius* ecology are scarce. Species of the genus were recorded inhabiting the bark of trees (Franz 1955). The only host plant records are for *P. echinus* and *P. singularis*, registered on *Cupressus* sp. (Cupressaceae) (Becker 1955).

Lacordaire (1869) designated *Moneilema inaequalis* Say, 1835 as the type species and the genus was monotypic at that time. In his description, Say (1835) mentioned: “*I place it in the present genus* [*Moneilema*], *although the approximation of the antennae is an obvious distinction*.” In erecting the genus *Phrynidius*, Lacordaire stated: “*Say hesitated to place this insect in Moneilema, with whom it has, in fact, nothing in common*.” Later, *Phrynidius echinus* and *Phrynidius singularis* were described by Bates (1880). All species of the genus are distributed only in the Mesoamerican region. The former, *P. echinus*, is distributed in Guatemala, Honduras, Costa Rica and Panama, while the latter, *P. singularis* is known from Mexico, Guatemala and Honduras (Tavakilian and Chevillotte 2020). *Phrynidius asper*, described by Bates (1885) five years later, is recorded from Guatemala, Honduras and Nicaragua (Tavakilian and Chevillotte 2020). *Phrynidius armatus*, distributed in Guatemala and Nicaragua, was described by Linsley (1933). Seven years later, *Phrynidius echinoides* was described by Breuning (1940) from Mexico. However, this record is probably incorrect, since the type locality is “Cerro Zunil”, which is located in Guatemala and not in Mexico (Selander and Vaurie 1962). After that, in 1954, *Phrynidius salvadorensis salvadorensis* and *Phrynidius salvadorensis montecristensis* were described by Franz, the former restricted to El Salvador and the latter to El Salvador and Honduras (Tavakilian and Chevillotte 2020). The most recently described species is *Phrynidius nayaritensis* (Heffern et al. 2018) only recorded from Mexico.

The most extensive study of the group was published by Breuning (1971), who reviewed the genus and provided a key for the species known at that time. In Breuning (1971), *Phrynidius* and *Parmenonta* Thomson, 1868 (currently equal to *Adetus* LeConte, 1852) were separated by the prominent antennal tubercles of the former. After Breuning’s revision, the genus has not been comprehensively studied, with most new distribution records published in recent years (Chemsak et al. 1980; Noguera and Chemsak 1996; Turnbow et al. 2003; Hovore 2006; Maes et al. 2010; Swift et al. 2010; Gutierrez and Noguera 2015; Audureau and Roguet 2018). In this work, we present four new species of the genus from Chiapas, Mexico, increasing the diversity of the genus to twelve species.

## Materials and methods

Photographs were taken with a Zeiss microscope with a Plan lens NeoFluar 2, 1×10.25 FWD 56. Measurements are given in mm and taken using an ocular micrometer 1.0× in the stereo Zeiss stereo Discovery microscope V8 FW. The types are deposited in the Colección Nacional de Insectos (**CNIN**), at the Instituto de Biología, Universidad Nacional Autónoma de México, México City, México.

## Results

### Cerambycidae Latreille, 1802

**Lamiinae Latreille, 1825**

**Apomecynini Thomson, 1860**

***Phrynidius* Lacordaire, 1869**

#### Phrynidius cristinae sp. nov.

Taxon classificationAnimaliaColeopteraCerambycidae

930B0EDA-4CF6-52EC-B74B-5D9089722F65

http://zoobank.org/236D5F2F-5BD0-4E76-BABA-74ABDE330738

Fig. 1A–C

##### Type material.

Holotype female: Mexico, Chiapas, Reserva El Triunfo, 12-Julio-1993, C. Mayorga. Approximate coordinates: 15°39'N, 92°48'W. COL.TIP-03711.

##### Diagnosis.

**Figure 1.**
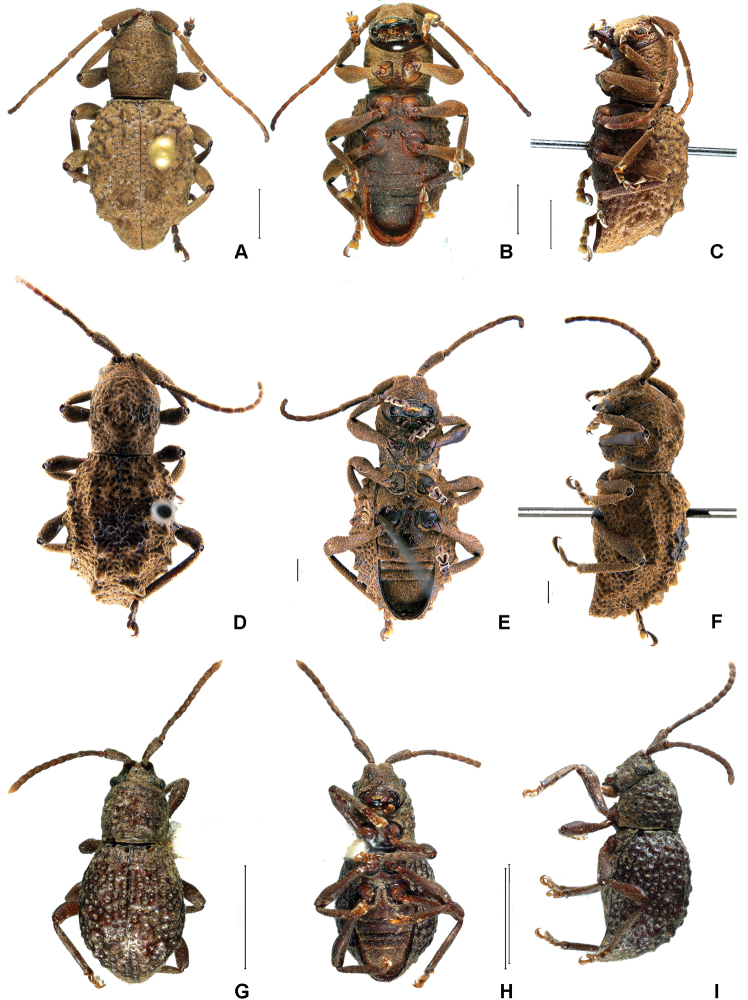
Dorsal, ventral and lateral view of three species of *Phrynidius***A–C***Phrynidius cristinae* sp. nov., holotype female **D–F***Phrynidius armatus* Linsley, allotype female **G–I***Phrynidius diminutus* sp. nov., holotype female. Scale bars: 2 mm (**A–C, G–I**), 1 mm (**D–F**).

This species is morphologically similar to *P. armatus* (Fig. 1D–F), but it is distinguished from it by the disposition of the elytral tubercles. In *P. armatus*, these are arranged in two longitudinal rows on the disc, one near the suture and the other near the elytral slope. In *P. cristinae* sp. nov., the tubercles are arranged as follows: on the basal half near the suture, three moderately prominent tubercles are arranged forming a triangle; on the apical half, there are five moderately prominent tubercles, with four of them forming a square.

##### Description.

***Female holotype*.** Length: 11.7 mm; width: 5.4 mm. Form moderately robust. Integument mostly dark brown, with labrum and apex of mandibles black; pubescence squamose, light brown, shining when exposed to light, dense, recumbent, minute, uniformly with small, curved and decumbent scale-shaped setae interspersed. *Head* with frons wider than long, slightly convex transversely, basal margin slightly angled medially, with suture extending from basal margin to back of the head; punctures coarse, deep, separated by spaces larger than their diameter; antennal tubercles short, vertical, contiguous basally, separated distally, upper margin glabrous, smooth, slightly projected interiorly; vertex slightly longitudinally depressed centrally, with punctures closer than those on frons; eye lobes widely separated on posterior margin, connected by two rows of ommatidia; lower eye lobes oval, wider than upper ones; genae convex, 1.8 times longer than length of lower eye lobes; postclypeus very narrow, longitudinally convex, clothed with thin, short setae and scarce long setae interspersed; anteclypeus narrow, glabrous, smooth and transversely convex; labrum with apical margin fringed with dense golden setae; antennae 0.88 times length of body, with dense pubescence and erect setae interspersed to almost the apex of fourth antennomere, remaining antennomeres with very fine pubescence that does not obscure integument, and some short and erect setae interspersed, scape slightly expanded to apex and slightly arched, antennomeres cylindrical, last antennomere slightly acuminate; antennal formula (ratio) based on length of the third antennomere: I = 1.00; II = 0.14; IV = 0.73; V = 0.41; VI = 0.38; VII = 0.32; VIII = 0.29; IX = 0.29; X = 0.26; XI = 0.35. *Thorax* Pronotum 1.06 times wider than long; subcylindrical, with apex slightly narrower than base; anterior margin oblique toward sides; posterior margin straight; sides slightly curved; disc convex, with five small, low tubercles placed on vertex of imaginary pentagon in the middle, another small, low tubercle on center of pentagon, and small protrusions placed around disc, giving integument rough appearance; punctures small, deep, separated by distance larger than their diameter. *Prosternum* length: 1.42 mm; width: 0.34 mm; short, 0.54 times width of procoxae, declivous; procoxal process arched, 0.5 times width of procoxae, apically widened with posterior margin straight, with deep punctures, contiguous to confluent, giving integument rough appearance on posterior half; mesosternum very short, strongly depressed; mesocoxal process 0.5 times width of mesocoxa, slightly widened apically, posterior margin notched centrally, with deep punctures, contiguous to confluent, giving integument rough appearance on posterior half; metasternum short, length equal to 0.6 times width of metacoxa, base strongly emarginate centrally, forming deep circular depression in conjunction with apex of first abdominal segment, and with small, deep depression on each side near internal angle of metacoxa. *Scutellum* small, triangular, with rounded apex, projecting up and clothed with pubescence. *Elytra* humeral width: 2.57 mm; elytral length: 6.11 mm; 1.3 times longer than wide; oval-shaped, with narrowest area posteriorly; strongly convex; sides deflexed, oblique, forming angle of 130° with horizontal line of abdomen; basal margin straight; apex rounded; with obtuse, slightly to moderately prominent, widely dispersed tubercles; on basal half, three tubercles moderately prominent near suture, forming triangle; on apical half, five moderately prominent tubercles, four forming square; punctures small, moderately deep, separated by distance larger than their diameter, evenly distributed (except on tubercles); pubescence dense, obscuring integument. *Legs* with femora moderately widened on apical half, with internal margin straight; tibiae straight with apex slightly widened; pubescence squamose, dense, with scale-shaped setae uniformly interspersed; tibiae with apex clothed with pale, thin, recumbent setae, and margin with golden, short setae; pro- and mesotibiae with sinus on hind third; tarsi with pale, thin setae dorsally, not obscuring integument, and ventral pads with pale-yellow setae. *Abdomen* with segments longitudinally convex; first segment 1.15 times longer than second, third and fourth segments of same length, each one 0.5 times length of first segment, last segment twice as long as first segment, strongly depressed on hind third and with apex rounded; pubescence dense, obscuring integument.

##### Etymology.

This species is dedicated to Cristina Mayorga, who collected the holotype, in recognition of her long career in entomology in Mexico.

#### Phrynidius diminutus sp. nov.

Taxon classificationAnimaliaColeopteraCerambycidae

13C2B998-F625-5F9B-80CF-F666144AEBC2

http://zoobank.org/580C67B0-0589-47D3-AC9D-1FFC70EB57D3

Fig. 1G–I

##### Type material.

Holotype female: Mexico, Chiapas, Municipio San Cristobal, Reserva Huitepec, 6-III-94, R. Jones, 4.2 HR *Q. rugosa*. Approximate coordinates: 16°45'N, 92°40'W. COL.TIP-03712.

##### Diagnosis.

This species is distinguished from the other species of *Phrynidius* by the absence of prominent elytral tubercles. The few tubercles present on the elytra are barely distinguishable in dorsal view, and are most distinct in lateral view, in which they are seen as blunt bumps. The other species of *Phrynidius* present easily distinguishable tubercles in lateral and dorsal view, either blunt or pointed.

##### Description.

***Female holotype*.** Length: 4.5 mm; width: 2.25 mm. Form small. Integument mostly dark brown, with antennae and legs brown, labrum, palpi, and tarsi light brown, margins of pronotum, elytra and coxal cavities fuscous, and apex of mandibles black; pubescence comprising small, arched, recumbent scale-shaped setae, interspersed uniformly, denser on legs. *Head* with frons longer than wide, slightly transversely convex, with median suture extending from half frons to vertex; punctures coarse, deep, almost contiguous; antennal tubercles short, vertical, contiguous basally, slightly separated distally, with fuscous upper margin, glabrous and smooth; eye lobes almost connected, separated slightly by a glabrous strip; lower eye lobes oval, wider than upper ones; genae slightly transversely convex, 1.5 times longer than lower eye lobes; anteclypeus narrow, glabrous, transversely convex; postclypeus narrow, longitudinally convex, with thin, short, yellow setae toward sides and scarce long setae interspersed; labrum strongly longitudinally convex, with apical margin fringed with dense golden setae; antennae 0.7 times body length, with dense, erect curved setae partially obscuring integument to almost middle of fourth antennomere, remaining antennomeres with fine semierect golden setae that does not obscure integument, scape slightly curved, antennomeres cylindrical; antennal formula (ratio) based on length of the third antennomere: I = 1.13; II = 0.17; IV = 0.52; V = 0.43; VI = 0.34; VII = 0.34; VIII = 0.34; IX = 0.26; X = 0.26; XI = 0.28. *Thorax* Pronotum 1.2 times longer than wide; subcylindrical; sides slightly curved, tapering apically; anterior margin strongly oblique toward sides; posterior margin straight; disc very transversely convex, with four small protrusions each placed on vertex of imaginary diamond centrally; punctures coarse, deep, contiguous, giving integument rough appearance. *Prosternum* length: 0.63 mm; width: 0.13 mm; short, slightly longer than width of procoxal process; procoxal process arched, 0.37 times width of procoxae, apically widened, with posterior margin straight; mesosternum very short, strongly depressed; mesocoxal process 0.37 times width of mesocoxae, slightly arched and widened apically, with posterior margin straight; metasternum short, length equal to half the width of mesocoxae. *Scutellum* triangular, slightly convex at apex. *Elytra* humeral width: 1.36 mm; elytral length: 2.73 mm; 1.2 times longer than wide, oval-shaped, with narrowest area posteriorly; strongly convex; sides deflexed, oblique, forming angle of 130° with horizontal line of abdomen; basal margin straight, slightly oblique toward suture; lateral margin bisinuate; apex rounded; with scattered, small, wide, blunt tubercles widely separated from each other, those located near base and sides smaller and gradually becoming larger toward suture and apex; with largest tubercles on base of apical third near suture; punctures coarse, contiguous, giving to the integument areolate appearance. *Legs* with femora moderately widened at apical half, with internal margin straight; tibiae with apical margin rounded, margined with golden erect setae and apical area only with erect golden setae; pro- and mesotibiae with sinus on hind third; tarsi almost glabrous dorsally and ventral pads with pale-yellowish setae. *Abdomen* with segments 2–4 longitudinally convex; first segment 1.2 times longer than second, third and fourth segments of same length and each one 0.5 times length of second segment, last segment twice as long as second segment, depressed laterally and apically, with rounded apex; with scale-shaped and straight setae interspersed uniformly, moderately dense, not obscuring integument.

##### Etymology.

The name of the species refers to its small size (4.5 mm).

#### Phrynidius jonesi sp. nov.

Taxon classificationAnimaliaColeopteraCerambycidae

EFAB60A2-2A67-524E-AD4F-C53458BB9918

http://zoobank.org/2F07BB1E-A9A2-4CB7-8F09-E5489F0EE6B8

Fig. 2A–C

##### Type material.

Holotype male: Mexico, Chiapas, Municipio Trinitaria, Cinco Lagos, Lagos de Montebello, 11-IX-1994, R. Jones. Approximate coordinates: 16°6'N, 91°40'W. COL.TIP-03713.

##### Diagnosis.

This species is distinguished from other species of the genus by the prominent elytral tubercles, with blunt, glabrous and black apex. The other species of *Phrynidius* with prominent tubercles have them clothed with pubescence.

##### Description.

***Male holotype*.** Length: 11.7 mm; width: 5.4 mm. Form moderately robust. Integument mostly dark brown to fuscous, with labrum, upper margin of antennal tubercles, scutellum, apex of elytral tubercles and first four tarsal segments black; pubescence squamose, light brown, shining when exposed to light, appressed, minute, uniformly interspersed with small, curved and almost decumbent scale-shaped setae. *Head* clothed with pubescence obscuring integument and setae interspersed; frons longer than wide, slightly transversely convex, with median suture extending to vertex; punctures coarse, deep, separated by twice their diameter; antennal tubercles low, vertical, contiguous at base, slightly separated distally, with upper margin glabrous, smooth and slightly projected interiorly; vertex slightly longitudinally depressed centrally, with punctures as on frons; eye lobes connected by glabrous strip; upper eye lobes tear-shaped, with widest part toward vertex, dorsally separated by 5 times its width; lower eye lobes oval, wider than upper ones; genae convex, two times longer than length of lower eye lobes; postclypeus narrow, convex longitudinally, with thin, short setae toward sides and scarce long setae interspersed; anteclypeus narrow, glabrous, transversely convex; labrum with apical margin fringed with dense golden setae; antennae 1.15 times longer than body, with dense pubescence and erect setae interspersed to almost apex of fourth antennomere, remaining antennomeres with very fine pubescence that does not obscure integument and some short and erect setae interspersed, scape slightly expanded toward apex and slightly curved, antennomeres cylindrical, last antennomere slightly acuminate; antennal formula (ratio) based on length of the third antennomere: I = 0.89; II = 0.10; IV = 0.78; V = 0.30; VI = 0.28; VII = 0.28; VIII = 0.26; IX = 0.26; X = 0.26; XI = 0.28. *Thorax* Pronotum 1.04 times longer than wide; subcylindrical, slightly tapering apically; anterior margin oblique toward sides; posterior margin straight; almost parallel-sided, with irregular margins; disc very convex, with prominent, subconical tubercle with apex directed backward, slightly ahead of midline; punctures coarse, deep, contiguous-confluent, giving integument rough appearance; dense pubescence obscuring integument. *Prosternum* length: 1.94 mm; width: 0.52 mm; short, equal length as width of procoxal process, apically oblique; procoxal process arched, 0.5 times width of procoxae, apically widened, with posterior margin straight; mesosternum very short, strongly depressed; mesocoxal process 0.4 times width of mesocoxae, slightly widened apically, with posterior margin straight; metasternum short, slightly shorter than width of mesocoxae, with narrow, curved transverse depression in hind third, extending on both sides from almost half to metepisternum. *Scutellum* triangular, slightly convex at apex, polished. *Elytra* humeral width: 2.94 mm; elytral length: 7.35 mm; 1.3 times longer than wide, oval-shaped, with narrowest area posteriorly; strongly convex; sides deflexed, oblique, forming angle of 130° with horizontal line of abdomen; basal margin straight, slightly oblique toward suture; apex rounded; with blunt tubercles ordered subserially from near base to almost apex, widely separated from each other, all tubercles with glabrous apex, those located near base and sides small and gradually becoming larger toward suture and apex, with largest tubercles on base of hind third near suture; punctures coarse, contiguous, moderately deep and evenly distributed (except on tubercles), giving integument areolate appearance; pubescence dense, obscuring integument. *Legs* with very dense pubescence, obscuring most of integument and scale-shaped setae uniformly interspersed; tibiae with apex glabrous, margined with golden, rigid and short setae; tarsi with pale and thin setae dorsally, not obscuring integument; ventral pads with pale-yellow setae. *Abdomen* with segments transversely convex; first segment 1.4 times longer than second, third and fourth segments of same length and each one 0.5 times length of second segment, last segment twice as long as second segment, with rounded apex; pubescence dense, obscuring integument and scale-shaped setae uniformly interspersed.

##### Etymology.

This species is dedicated to Robert Jones, who collected the holotype, in recognition of his contribution to entomology in Mexico.

#### Phrynidius tuberculatus sp. nov.

Taxon classificationAnimaliaColeopteraCerambycidae

5919FB02-C735-5C62-9032-EDA37E8215DA

http://zoobank.org/44281CF5-78E1-4CE1-B8F1-EB973069DE6B

Fig. 2D–F

##### Type material.

Holotype male: Mexico, Chiapas, Jaltenango, El Triunfo, 23-VIII-1996, Col V. H. Toledo. Approximate coordinates: 15°52'N, 92°43'W. COL.TIP-03714.

##### Diagnosis.

This species can be recognized from other species of the genus by the very prominent, subconical, oblong mid tubercle on pronotum, and the prominent tubercles aligned in two rows on the elytra, with tubercles located on the posterior half, being the most prominent and pointed on apex. The remaining species of *Phrynidius* with prominent tubercles on the posterior half of the elytra have them with blunt, not pointed apex.

##### Description.

***Male holotype*.** Length: 8.9 mm; width: 5.2 mm. Form small, moderately robust; integument dark brown, except postclypeus, antennae (except scape), coxae and trochanters lighter brown; pubescence squamose, light brown, prostrate, dense, obscuring integument, with small, curved and recumbent scale-shaped setae interspersed. *Head* with frons longer than wide, slightly transversely convex, with basal margin beveled and medially angled; punctures coarse, deep, separated by twice their diameter; antennal tubercles low, contiguous basally, slightly separated distally; eye lobes widely separated on posterior margin, connected by two rows of ommatidia; lower eye lobes oval, wider than upper ones; genae convex, two times longer than length of lower eye lobes; anteclypeus narrow, glabrous, transversely convex; postclypeus narrow, longitudinally convex, with thin, short setae toward sides and scarce long setae interspersed; labrum with apical margin fringed with dense golden setae; antennae 0.99 times body length, with minute dense pubescence, partially obscuring integument, becoming finer to distal antennomeres, with short recumbent setae interspersed, scape thickened, slightly expanded to apex and slightly curved, antennomeres cylindrical, last antennomere slightly acuminate; antennal formula (ratio) based on length of the third antennomere: I = 1.10; II = 0.14; IV = 0.71; V = 0.42; VI = 0.38; VII = 0.38; VIII = 0.35; IX = 0.33; X = 0.33; XI = 0.42. *Thorax* Pronotum 1.32 times longer than wide; subcylindrical, with base slightly wider than apex; anterior margin oblique toward sides; posterior margin straight; parallel-sided with irregular margins; disc transversely convex; mid tubercle very prominent, subconical, oblong, extending from base of apical fifth to apex of basal fifth; punctures coarse, deep, contiguous-confluent, giving integument rough appearance; pubescence dense, obscuring integument. *Prosternum* length: 1.23 mm; width: 0.42 mm; short, depressed transversely in middle area; procoxal process arched, 0.6 times width of procoxae, apically widened, with posterior margin straight; mesosternum depressed, slightly transversely convex, short, 0.5 times width of mesocoxae; mesocoxal process oblique, curved, 0.5 times width of mesocoxae, longitudinally depressed on posterior half, slightly widened apically, with posterior margin angled inward, with punctures coarse, deep, giving integument rough appearance; metasternum short, slightly shorter than width of mesocoxae, with deep circular depression that comprises metasternum base and apex of first abdominal segment. *Scutellum* small, triangular, with glabrous apex, rounded, with small obtuse tubercle. *Elytra* humeral width: 1.92 mm; elytral length: 5.19 mm; 1.3 times longer than wide, oval-shaped, with narrowest area posteriorly; strongly convex; sides deflexed, oblique, forming angle of 130° with horizontal line of abdomen; basal margin straight, slightly oblique toward suture; apex rounded; with prominent tubercles aligned in two rows, one row on the disc, parallel to suture, which extend from base of first seventh to slightly behind middle part, the other row follows contour of elytral slope from base of first seventh to almost suture; first row consists of two discontinuous tubercles, first subconical and obtuse, second elongated and twice length of first, forming crest, second row consists of three conical and obtuse tubercles, the first separated from the other two by twice of its diameter, the other two more prominent of all, separated by space smaller than their diameter, slightly pointed, in addition, just back and to the sides of suture, one small and less prominent tubercle on each side, scattered between prominent tubercles, there are small, obtuse tubercles; punctures coarse, contiguous, moderately deep and evenly distributed (except on tubercles), giving integument rough appearance; pubescence dense, partially obscuring integument. *Legs* with femora clavate in apical half, with internal margin straight; tibiae straight with apex slightly widened; pubescence squamose and dense, obscuring most of integument and with scale-shaped setae uniformly interspersed, except tibiae that have glabrous apex, margined with golden, rigid and short setae; protibiae with sinus on the hind third; tarsi with pale and thin setae dorsally, not obscuring integument, and ventral pads with pale-yellow setae. *Abdomen* with segments transversely convex; first segment 1.1 times longer than second, third and fourth segments of the same length, each one 0.6 times length of second segment, last segment almost twice as long as second segment, with rounded apex, margined with long golden setae; pubescence dense, obscuring integument and scale-shaped setae uniformly interspersed.

**Figure 2.**
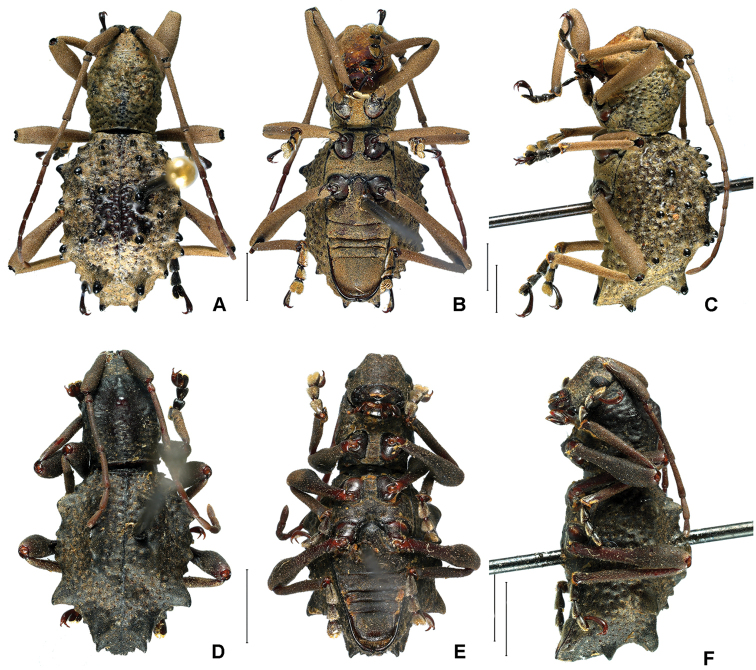
Dorsal, ventral and lateral view of two species of *Phrynidius***A–C***Phrynidius jonesi* sp. nov., holotype male **D–F***Phrynidius tuberculatus* sp. nov., holotype male. Scale bars: 2 mm.

##### Etymology.

The name of this species refers to the conspicuous tubercles on the elytra.

#### Key to the species of *Phrynidius*

**Table:** 

1	Gena about 1.5 times longer than length of lower eye lobe	**2**
–	Gena about 2 times longer than length of lower eye lobe	**3**
2(1)	Third antennomere longer than scape, surpassing the base of pronotum; pronotum with tubercles. Guatemala	***P. echinoides* Breuning**
–	Third antennomere shorter than scape, not exceeding the base of pronotum; pronotum not tuberculate. Mexico	***P. diminutus* sp. nov.**
3(1)	Pronotum with gibbosity or prominent middle tubercle	**4**
–	Pronotum without gibbosity or prominent middle tubercle	**8**
4(3)	Metafemora elongated or slightly thickened toward the apex; elytra with blunt tubercles on apex	**5**
–	Metafemora apically clavate; elytra with two rows of tubercles, those located on the posterior half are very prominent and sharpened apically. Mexico	***P. tuberculatus* sp. nov.**
5(4)	Elytral tubercles moderately prominent, completely covered with pubescence	**6**
–	Elytral tubercles prominent, with glabrous apex. Mexico	***P. jonesi* sp. nov.**
6(5)	Scape as long or shorter than third antennomere	**7**
	Scape longer than third antennomere. Guatemala, Honduras and Nicaragua	***P. asper* Bates**
7(6)	Elytra with abundant tubercles, with slightly wide base and more prominent in the posterior half. Mexico	***P. nayaritensis* Heffern, Nascimento and Santos-Silva**
–	Elytra with moderately abundant tubercles, with wide base and similar proportions in both anterior and posterior half. Mexico, Guatemala and Honduras	***P. singularis* Bates**
8(3)	Scape equal in length or shorter than third antennomere	**9**
–	Scape longer than third antennomere	**10**
9(8)	Scape as long as third antennomere. Mexico	***P. cristinae* sp. nov.**
–	Scape distinctly longer than third antennomere. Guatemala, Honduras, Costa Rica and Panama	***P. echinus* Bates**
10(8)	Elytra with tubercles on lateral and apical slopes, where they are slightly more prominent	**11**
–	Elytra without tubercles on lateral or apical slopes. Mexico	***P. inaequalis* Say**
11(10)	Elytral disc with moderately prominent tubercles forming a circle, and a line of three prominent tubercles on each side of the apical slope. Guatemala and Nicaragua	***P. armatus* Linsley**
–	Elytral disc with a few slightly prominent tubercles irregularly arranged, with prominent tubercles on the apical slope, irregularly arranged or in a line of two. El Salvador and Honduras	***P. salvadorensis* Franz**

## Supplementary Material

XML Treatment for Phrynidius cristinae

XML Treatment for Phrynidius diminutus

XML Treatment for Phrynidius jonesi

XML Treatment for Phrynidius tuberculatus
