# ﻿Addenda and corrigenda: Gutiérrez N, Toledo-Hernández VH, Noguera FA (2020) Four new species of *Phrynidius* Lacordaire (Coleoptera, Cerambycidae, Lamiinae) from Mexico with an identification key for the genus. ZooKeys 1000: 45–57. https://doi.org/10.3897/zookeys.1000.56757

**DOI:** 10.3897/zookeys.1089.80564

**Published:** 2022-03-18

**Authors:** Nayeli Gutiérrez, Víctor H. Toledo-Hernández, Sergio Devesa, Eduardo Rafael Chamé-Vázquez, Felipe A. Noguera

**Affiliations:** 1 Division of Invertebrate Zoology, Richard Gilder Graduate School, American Museum of Natural History. Central Park West & 79; 2 th; 3 St, New York, NY 10024, USA; 4 Centro de Investigación en Biodiversidad y Conservación, Universidad Autónoma del Estado de Morelos, Av. Universidad 1001, Col. Chamilpa, Cuernavaca, Morelos 62209, Mexico; 5 La Iglesia, 4, San Vicente do Grove, 36988 Pontevedra, Galicia, Spain; 6 Ecología de Artrópodos y Manejo de Plagas, El Colegio de la Frontera Sur, Carretera Antiguo Aeropuerto Km. 2.5, CP 37000, Tapachula, Chiapas, Mexico; 7 Estación de Biología Chamela, Instituto de Biología, Universidad Nacional Autónoma de México, Apartado Postal 21, San Patricio, Jalisco 48980, Mexico

**Keywords:** Biodiversity, Central America, longhorn beetles, taxonomy

## Abstract

After describing *Phrynidiusjonesi* Gutiérrez, Toledo & Noguera, 2020 (Coleoptera, Cerambycidae, Lamiinae), the authors had the opportunity to study a conspecific individual of this species and recognize that the holotype had been erroneously determined as a male when in fact it was a female. Here, we rectify this error and provide morphological information for the identification of both sexes. Additionally, we record *Phrynidiusarmatus* Linsley, 1933 from Chiapas, Mexico. Finally, we document *P.cristinae*Gutiérrez et al. 2020 feeding on a fungus, which represents the first record for any species of the genus *Phrynidius* with this adult feeding habit.

## ﻿Introduction

Knowledge of the genus *Phrynidius* Lacordaire, 1869 (Coleoptera, Cerambycidae, Lamiinae) is restricted to taxonomy and distribution, while host plant information is known for only one of its species. Fortunately, the collection of new specimens represents an opportunity to increase our knowledge about other aspects of the natural history of this group. This is the case for *P.jonesi* Gutiérrez, Toledo & Noguera, 2020 and *P.cristinae* Gutiérrez, Toledo & Noguera, 2020, both from Chiapas, Mexico, as well as for *P.armatus* Linsley, 1933 from Guatemala, of which we had the opportunity to study recently collected specimens. Following its description, an additional specimen of *P.jonesi* was collected at the type locality. This new specimen allowed for the reevaluation of our original description. In this note, we rectify the sexual identity of the holotype of *P.jonesi*, describe the sexually dimorphic characters of the species, and provide new information about its known hosts. In addition, we record for the first time *P.armatus* from Chiapas and adult feeding habits for *P.cristinae*.

## ﻿Materials and methods

Photographs of *P.jonesi* were taken with a Canon EOS 5D Mark III DSLR equipped with a Canon MP-E 65 mm f/2.8 1–5× macro lens objective and automatically controlled with a Cognisys Stackshot. Photographs were focus stacked with the Zerene Stacker AutoMontage software and processed on Capture One 21. Photographs of the habitat were taken with an iPhone 8. The specimen is deposited in the Sergio Devesa Personal Collection (**SDPC**), Pontevedra, Spain. Photographs of *P.cristinae* were taken with a Canon 70D camera equipped with a Canon 60 mm, f /2.8 macro lens. Specimens of *P.cristinae* and *P.armatus* are deposited in Colección de Insectos de la Universidad de Morelos (**CIUM**), Morelos, Mexico and Colección de Insectos Asociados a Plantas Cultivadas en la Frontera Sur (**ECO-TAP-E**), Chiapas, Mexico.

## ﻿Errata

*Phrynidiusjonesi* Gutierrez et al., 2020

**p. 51, ninth line**: “Male holotype” should read “Female holotype”.

*Phrynidiusarmatus* Linsley, 1933

**p. 46, seventeenth line**: “distributed in Guatemala and Nicaragua” should read “distributed in Guatemala, Mexico and Nicaragua”.

**p. 55, thirty-seventh line**: “Guatemala and Nicaragua” should read “Guatemala, Mexico and Nicaragua”.

## ﻿Additions

***Phrynidiusjonesi* Gutiérrez, Toledo & Noguera, 2020**

Fig. 1A–D

**Sex**: Male. **Locality**: Mexico, Chiapas, Municipio de La Trinitaria, Lagunas de Montebello, 08-I-2019, 16°06'27.55N, 91°42'39.17W. S. Devesa leg.

**Figure 1.**
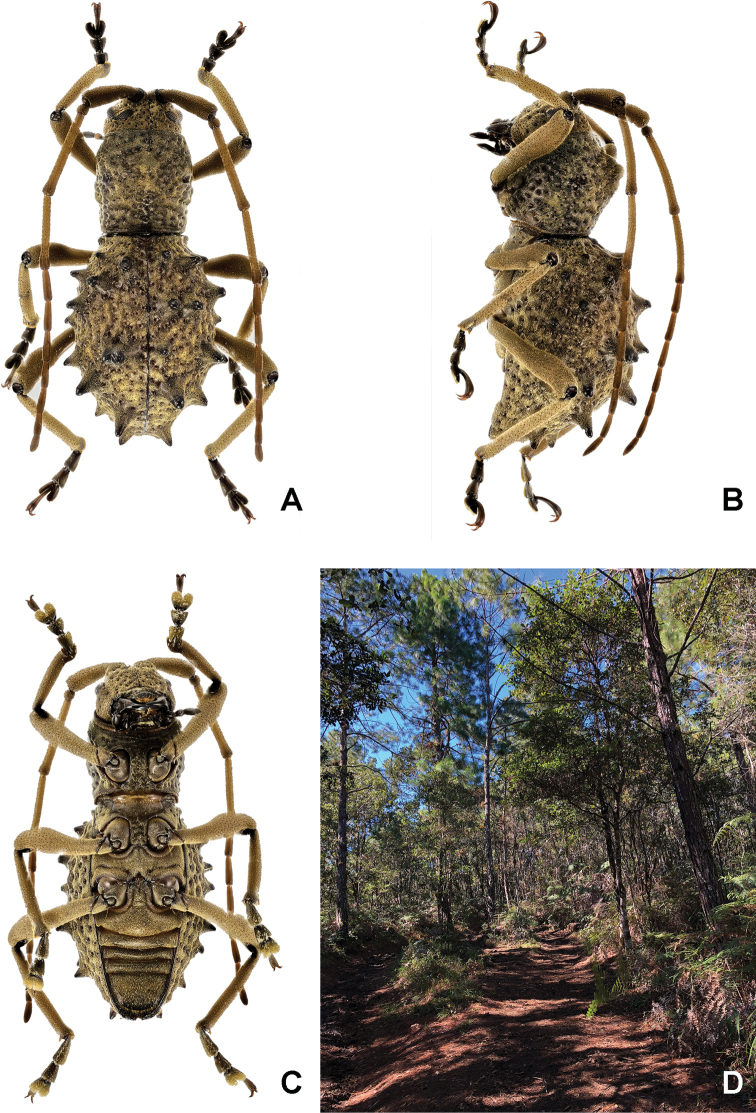
*Phrynidiusjonesi* Gutiérrez, Toledo & Noguera, 2020: **A–C** male: Dorsal, ventral, and lateral views **D** habitat where the specimen was collected.

The male differs from the female type in the following characters: smaller body size (10.2 vs. 11.7 mm); antennae longer relatively to body length (1.21 times longer than body, 1.15 times longer than body in female); antennal formula (ratio, based on length of the third antennomere) I = 0.88, II = 0.12, IV = 0.84, V = 0.36, VI = 0.32, VII = 0.32, VIII = 0.28, IX = 0.28, X = 0.28, XI = 0.28; abdomen more slender and elongated; last abdominal segment shorter, uniformly convex to the apical margin; apex almost glabrous and margin with a fringe of setae (in female, last abdominal segment more convex, with curvature not extending to apical margin, which is more flattened than in male).

The larva of *P.jonesi* was collected under bark of *Pinusoocarpa* Schiede ex Schltdl. (Pinaceae). This constitutes a new host record for the genus since the only host plant of *Phrynidius* species known to date was *Cupressus* sp. (Cupressaceae) (Becker 1955). The larva was collected on January 8^th^ and the adult emerged on June 28^th^ of the same year. This indicates that the development from larva to adult in this specimen lasted at least 6 months and 20 days. It is important to mention that the larva was kept in artificial conditions, therefore this developmental time could differ from development in the conditions of its habitat.

***Phrynidiusarmatus* Linsley, 1933**

**Sex**: Male. **Locality**: México: Chiapas, Municipio Villacorzo, Ejido Sierra Morena, REBISE. 15-VI-2016, 1746 msnm, 16°08'16.88"N, 93°36'19.87"W (4 specimens). E.R. Chamé-V. col. **New state record**.

Male (9.7 mm) slightly longer than holotype (9.0 mm). Specimens of *P.armatus* were collected in a cloud forest with a high abundance of *Tillandsiausneoides* (L.) L. (Bromeliaceae). The specimens were collected using a beating sheet, which was placed under the vegetation (2 to 3 m high) and beaten with a pole. The type series was collected in Santa Ilena (probably Santa Elena), Guatemala, and the species was later recorded from Veracruz, México, and Selva Negra Mountain Resort, Nicaragua (Linsley 1933; Noguera and Chemsak 1996; Audureau and Roguet 2018). The specimens reported here were collected in Sierra Morena, Chiapas, which is part of the Sierra Madre of Chiapas, a mountain range connected with Chimaltenango, Guatemala. Both localities are in cloud forests.

***Phrynidiuscristinae* Gutiérrez, Toledo & Noguera, 2020**

Fig. 2A–D

**Sex**: Male. **Locality**: México: Chiapas, Municipio Villacorzo, Ejido Sierra Morena, REBISE. 03-VIII-2016, 1746 msnm, 16°08'16.88"N, 93°36'19.87"W. E.R. Chamé-V. col. (2 specimens).

**Figure 2.**
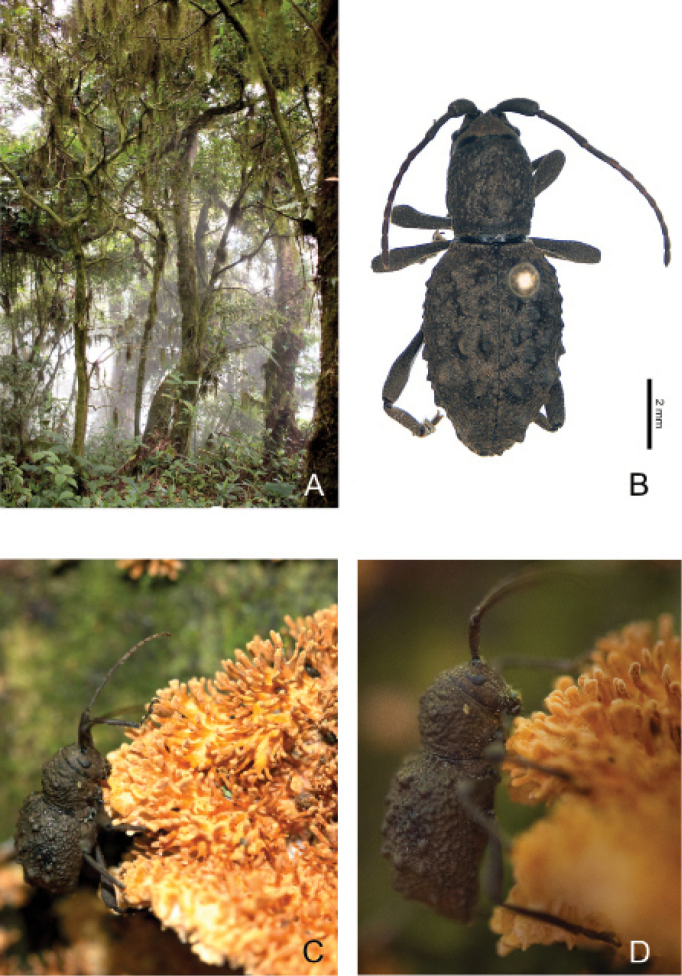
*Phrynidiuscristinae* Gutiérrez, Toledo & Noguera, 2020: **A** habitat where the specimen was collected **B** dorsal view of male **C, D** specimen feeding on *Echinoporiaaculeifera*.

On August 3, 2016 at 12:12 pm a specimen of *P.cristinae* with integument darker than that of the holotype was observed eating the pileus ornamentation of a specimen of *Echinoporiaaculeifera* (Berk. & M.A. Curtis) Ryvarden, 1984 (Schizoporaceae). This fungus was found in a rotten log, which had a conglomerate of several individuals of this species. *Phrynidius* species had only been associated with conifers (Cupressaceae and Pinaceae, recorded here), and no information was known about their feeding habits until now.

This is an interesting result, since only a few species of cerambycids have been recorded as fungal feeders as adults (Craighead 1923; Duffy 1953; Haack 2017; Michalcewicz 2002). Our findings emphasize the importance of observations in the field for a better understanding of the natural history of *Phrynidius*.
